# Tapping the Vast Potential of the Data Deluge in Small-scale Food-Animal Production Businesses: Challenges to Near Real-time Data Analysis and Interpretation

**DOI:** 10.3389/fvets.2017.00120

**Published:** 2017-09-06

**Authors:** Flavie Vial, Andrew Tedder

**Affiliations:** ^1^Epi-Connect, Skogås, Sweden; ^2^Department of Ecology, Environment and Plant Sciences, University of Stockholm, Stockholm, Sweden

**Keywords:** antimicrobial usage, food-animal production, agribusiness, digitization, farm data

## Abstract

Food-animal production businesses are part of a data-driven ecosystem shaped by stringent requirements for traceability along the value chain and the expanding capabilities of connected products. Within this sector, the generation of animal health intelligence, in particular, in terms of antimicrobial usage, is hindered by the lack of a centralized framework for data storage and usage. In this Perspective, we delimit the 11 processes required for evidence-based decisions and explore processes 3 (digital data acquisition) to 10 (communication to decision-makers) in more depth. We argue that small agribusinesses disproportionally face challenges related to economies of scale given the high price of equipment and services. There are two main areas of concern regarding the collection and usage of digital farm data. First, recording platforms must be developed with the needs and constraints of small businesses in mind and move away from local data storage, which hinders data accessibility and interoperability. Second, such data are unstructured and exhibit properties that can prove challenging to its near real-time preprocessing and analysis in a sector that is largely lagging behind others in terms of computing infrastructure and buying into digital technologies. To complete the digital transformation of this sector, investment in rural digital infrastructure is required alongside the development of new business models to empower small businesses to commit to near real-time data capture. This approach will deliver critical information to fill gaps in our understanding of emerging diseases and antimicrobial resistance in production animals, eventually leading to effective evidence-based policies.

## Introduction

The food-animal production sector, particularly in industrially developed countries, is evolving into a more data-driven ecosystem in which data are adding value not only to the business process but also to the entire food supply chain (1). This transformation is not only driven by technology but also by an increasing requirement for traceability and accountability initiated by regulatory frameworks (2), which can differ between countries, or directly by consumers (3). The use of antimicrobials in this sector, for both disease treatment and, in some countries, growth promotion, is one of the areas directly affected by this evolution, with usage in livestock projected to increase by 67% by 2030 (4). Monitoring the volume of antimicrobial medicines used, and understanding under which conditions they are administered, are essential for identifying possible risk factors that could lead to the development and spread of antimicrobial resistance in animals. In many developing countries, these concepts are still at a very primitive level where one can still buy antibiotics without prescription. In fact, even in regions with clear guidelines on usage recording [e.g., in the EU, usage of critically important antimicrobials must be recorded (5)], a data gap persists (6). In many cases, this data gap does not result from the farmers failing to record the treatments they administer to their animals but from the fact that the data are often not digitized, nor centrally collected and are thus not amenable, or accessible for analysis in a way that allows decision-makers to utilize them.

In industrial countries, large food-animal production businesses have embraced digitization and manage their core business processes with enterprise resource planning, mediated by software and technology, in real-time. At the other end of the spectrum, small businesses, which tend to be made up of independent smallholders and family-operated businesses, are still faced with the challenges of storing, managing, and predominantly extracting value from the data they generate in a cost-effective manner. Revealing actionable animal health and business insights from data requires large mobilizations of technologies, infrastructure, and expertise, which are often too elaborate and costly for small-scale food-animal production businesses.

**Figure 1:**
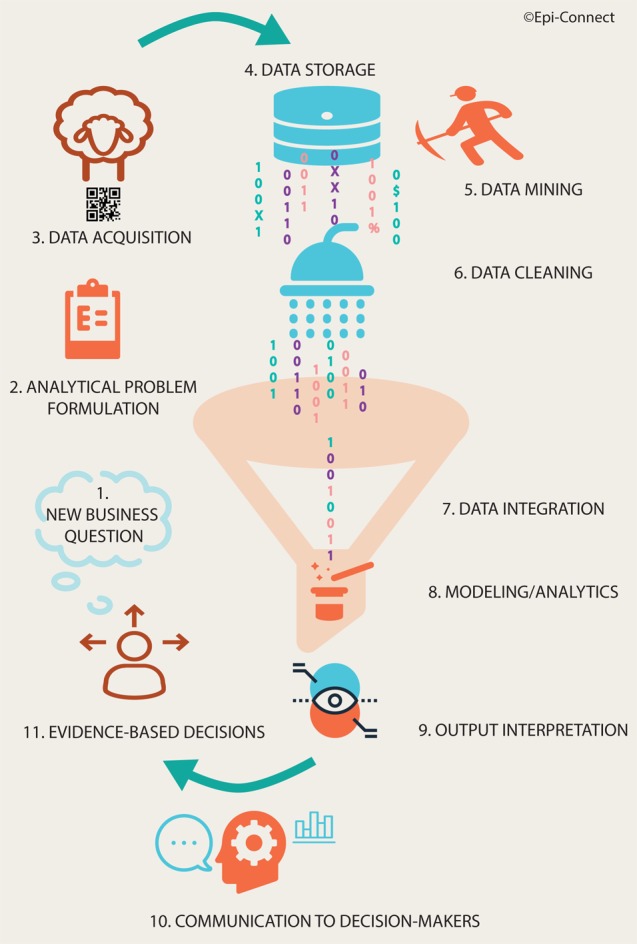
Cycle of processes necessary to generate actionable animal health intelligence for decision-makers.

With increasing demand for animal protein for human consumption resulting in an increase in livestock number in low-/middle-income countries, coupled to a shift in production toward large-scale intensive practices in middle/high-income countries (7), data management, from the farm-level up, is increasingly pertinent in today’s livestock value chain. We identified 11 processes taking place between the time a decision-maker, either farm- or office-based, formulates business questions and the moment they can take evidence-based decisions (Figure 1). In this Perspective paper, we explore processes 3 (digital data acquisition) to 10 (communication to decision-makers) in more depth, with a focus on the inherent challenges to the timely generation of animal health intelligence and how they disproportionally affect small-scale food-animal production businesses. While this perspective focuses on one particular type of agribusiness, food-animal production, many of the challenges and methodological solutions will also be applicable to other types and within other geopolitical jurisdictions.

## Data Acquisition and Storage (Processes 3–4)

The important question is not who captures the data, but how are they captured? Automated digital data capture increases data quality, by reducing bias in data entry, and volume while enabling the farmer to dedicate more time to animal care and maximizing returns. Today, a large proportion of agri-food data are collected through sensors and robots at all production scales (8) (e.g., milking machines), often incorporated in the “Internet of Animal Health Things” (9), a network of objects that communicate with each other and with computers through the Internet. This automation of data collection raises issues about data governance and the companies that commercialize data recording products, for example, questions regarding data ownership, data access rights, and potential lock-in effects (e.g., a farmer not being able to migrate their historical data if they move to another supplier) (10).

However, animal health and drug usage data are seldom collected digitally in small businesses. One important contributing factor to this is the absence of a suitable recording platform (e.g., an App), which would offer user-friendly, dynamic, and editable real-time data acquisition. This does not mean that data on animal health and drug usage are scarce. To the contrary, these data exist often in the form of paper logbooks, but only a relatively small proportion of these data are readily automatable or exploitable. Missing metadata or ambiguous units of measurements, for example, contribute to the data being unfit for the derivation of intelligence. New recording platforms must be developed with the needs and constraints of small businesses in mind and must adhere to the FAIR data approach (11), i.e., capture data that are

- *F*indable (by both humans and computer systems),
- *A*ccessible (stored for long-term use; Open Access when possible),
- *I*nteroperable (see next section),
- *R*eusable.

Concerns about data security coupled to an often poor rural digital infrastructure (e.g., lack of access to reliable high-speed internet) result in many small businesses storing their data locally. This hinders data accessibility and interoperability as on-farm data storage capabilities may not be adequate to cope with the volume of data continuously being generated or may be weak in terms of security protocols (i.e., data corruption).

Furthermore, data on drug usage should not only be stored for compliance purposes but perhaps more importantly to generate animal health intelligence and herd management insights. To this end, increasing the size of the dataset used to generate such intelligence, by aggregating the data over several businesses, will add additional value to the data generated by each individual business. Farm data communities represent one way forward in the digital transformation of small businesses. These communities, such as Data Linker^1^ in Australia/New Zealand, are increasingly being formed by farmers with a desire to take control over their data by choosing how they are shared in a way that may create opportunities for financial gains.

## Data Mining, Cleansing, and Integration (Processes 5–7)

Farm data communities may receive the support of farm data aggregators. These aggregators leverage the active participation of the data communities in order to (a) aggregate their data for the derivation of actionable intelligence and/or (b) provide commercial services to other market stakeholders (e.g., feed companies) while ensuring maximum financial return to the businesses generating the data. However, both functions are not trivial as a large proportion of the data collected in agribusinesses is unstructured, i.e., it is text-heavy and seldom stored in relational databases. As such, it is necessary to preprocess the data by

- extracting from that large data pool, the data subset that is relevant to the business question asked (data mining);
- correcting, or removing, corrupt or inaccurate records and articulate the data subset(s) in a standard and structured form (data cleaning);
- combining data subsets residing in different sources to ultimately provide the users with a unified view (data integration).

Automated digital farm data exhibit properties (e.g., variety, and in some cases, volume and velocity), which can prove challenging to its near real-time preprocessing in a sector that is lagging behind others in terms of computing infrastructure and investments in digital technologies. To mine and clean data, the right technology needs to be in place to go through the volume of data and access the level of detail needed, all at high speed. This necessitates upgrading to more powerful hardware, turning to a grid computing approach, where machines are used in parallel to solve a problem more rapidly, or to a cloud computing approach.

Data interoperability between agribusinesses is still very poor, with the characteristically amorphous data typically lacking any binding information. It becomes necessary to apply semantic technology, such as control vocabularies (ontologies) and standards (e.g., agroXML^2^), to provide anchors to help interoperate and link across data. More specific information on the application of semantic technology to animal health data can be found in Ref. (12). Ideally, such a data annotation scheme would be built into the tools designed to capture the data, so that this is done automatically.

## Modeling and Analytics (Process 8)

Mere accumulation of data without any relevant output is both costly and useless. Data must provide timely and actionable animal health and business insights, and as such, careful plans regarding how the data will be analyzed and for what purpose must be made before the data acquisition stage. Modeling and analytics is about extracting important common features across many subpopulations even when large individual variation exists. The outputs from this process can be descriptive in nature; prescriptive (e.g., provide recommendations for improvement of a process); or predictive (13).

Digital data are characterized by high dimensionality (a lot of random variables) and large sample size features, which raise the following three analytical challenges (14). High dimensionality brings (1) noise (error) accumulation; (2) spurious correlations; and (3) incidental endogeneity (when many unrelated covariates incidentally correlate with the residual noises). High dimensionality combined with large sample size creates issues such as heavy computational cost and algorithmic instability (how a machine-learning algorithm is perturbed by small changes to its inputs). Finally, large samples aggregated from multiple sources at different time points using different technologies create issues with experimental variation (e.g., data collected under different and potentially non-comparable settings) and statistical biases (e.g., differences between an estimator’s expected value and the true value of the parameter). Some of these challenges can be overcome through the development of more adaptive and robust statistical procedures; others rely on the analyst’s ability to correctly infer based on the data and their limitations. The latter is particularly important in an era in which software such as Tableau^3^ or IBM Watson^4^ have “democratized” data modeling and visualization, allowing individuals without a background in data science to easily create data summary products (with the aim of deriving business intelligence) without a thorough understanding of statistics and inference (also see point below).

Methodological solutions exist for the reconciliation of data privacy concerns in respect to market competition and regulatory groups accessing the data with the release of sensitive data for analysis. For example, remote analysis infrastructures such as DataSHIELD^5^ at Bristol University allow for pooled analyses without the need to access individual level data. Differential privacy addresses the paradox of maintaining individual anonymity, while increasing the understanding of a population (15) through the application of hashing (turning data into a unique string of random-looking characters), subsampling, and noise injection (adding random data to obscure sensitive personal information) techniques. Finally, data can be fed into complex statistical models to create synthetic data that are statistically identical (i.e., have the same statistical properties), but not an exact replica of the original data (16). Any query that can be asked of the original sensitive data can also be asked of the synthetic data, with the added benefit that the latter does not hold any personal information so that it cannot be traced back to any individual.

## Output Interpretation and Communication (Processes 9–10)

Results from the data analysis and modeling stage need to be presented, verbally or visually, to decision-makers in a way that allow them to extract animal health intelligence. This process exemplifies the need for context. It is not enough for the quantitative analyst to be proficient with numbers; they must possess adequate domain expertise, i.e., an understanding of where the data come from, of the audience that will be consuming the analytical output, and how that audience will interpret these insights. There is a need to train more analysts with livestock and veterinary public health domain expertise: experts who can not only interpret the underlying statistics but also talk to the stakeholders in a way they can understand.

Data providers and knowledge users (if different entities) should put in place data use agreements, which clearly lay out the terms and conditions under which the knowledge derived from the data can be communicated and disseminated. Communication of any identifying or other sensitive information in an open forum can significantly damage a producer’s livelihood and his/her reputation within the industry. With a strong data governance strategy in place, both negative and positive feedback to the data providers is to be encouraged in order to maintain or increase data quality, and hence data value, and ensure continued motivation and long-term data-driven business partnerships.

## Discussion

First and foremost, governments must provide or encourage (private) investments in rural digital infrastructure. Not only can these investments be expected to contribute to the resilience of rural communities, they will also help address policy challenges in a wide range of areas, including agriculture (17). For example, in the European Union, the new Animal Health Law [Regulation (EU) 2016/429]^6^ stipulates that farmers “should therefore maintain up-to-date records of information which is relevant for assessing the animal health status, for traceability and for an epidemiological enquiry in the event of the occurrence of a listed disease. Those records should be easily accessible to the competent authority.” While records in paper form are still legal (with the exception of some records, like animal identification, which must be electronic), the move is toward increasing the proportion of records kept digitally in the coming years. For mandatory data capture and submission, we argue that regulatory authorities have a duty to invest and develop safe data capture and storage protocols to maximize compliance and data quality.

The use of digital data products for animal health and business insights comes at a cost, albeit one which larger food-animal production businesses are able to afford with the anticipation of higher returns. Small businesses, however, face challenges related to economies of scale given the high price of equipment (e.g., for digital data capture or data storage) and services (e.g., data modeling and interpretation) related to data. So, how do we address this digital “power asymmetry” (18) between small and large agribusinesses?

One way to rebalance this power asymmetry could be through open-source data, and publicly funded analytical tools, for use in the public domain, which rival those of large agribusinesses in terms of complexity and innovation (18). Another solution could be to foster the use among small businesses of cloud computing technologies, i.e., buying information and communication technologies (ICT) as a service. Not only would this allow small agribusinesses to overcome some of the barriers associated with the high fixed costs of ICT investment (and its lock-in effect), it also allows them to switch more rapidly to newer/better technologies as the old ones become obsolete (17). We personally favor the latter and believe that the increasing number of small agribusinesses forming new, or joining existing farm data communities, as well as the advent of farm data aggregators provide the right conditions for this much-needed change in how ICT is done in this sector. Regardless of the choice of publicly funded analytical tools or buying ICT as a service, more should be done by knowledge users (e.g., regulatory authority or food standard scheme) to communicate to data producers (the agribusinesses) that data on their own, without the correct analysis and interpretation, are almost worthless. Data overload, in which enormous amount of data on animal monitoring and production are collected and left idle on a business’ IT system, does not produce any intelligence, which can aid decision-maker unless it goes through the right pipeline (steps 5–10).

Perceived digital security risks may constitute a barrier to the adoption of these solutions. A secure digital agri-food chain is a shared responsibility as some risks will be displaced outside an agribusiness’ span of control, highlighting the need for data governance strategies throughout the chain: what data are warehoused? Accessed? Analyzed? And by whom? These questions must be answered on a case-by-case basis, taking the needs and requirements of the businesses entering these data transactions. Attention must also be paid to questions of intellectual property. Ownership generally lies in who stores and controls the value of the data. However, isolated large datasets frequently hold little value; it is the combination of these data with that of quantitative analytics that creates the value (13), raising the questions of who owns the primary, secondary, or even the tertiary uses of the data? We feel that most people in the agri-sector do not hold the answers to these critical questions. Whether this is a result of the legal framework lagging behind the data revolution or a lack of transparency regarding the intellectual property, copyright and other ownership-like protections, small agribusinesses generating data fall under is unclear.

The animal health value chain has traditionally been a closed business ecosystem built on transactional relationships. This has resulted in knowledge being compartmentalized and in inefficient resource utilization. Today, this value chain is rapidly evolving with many new participants (e.g., feed companies, pharmaceutical companies) redefining the industry. Furthermore, the increasing capabilities of smart, connected products not only reshape competition within the industry but also expand the industry boundaries. As a result, we argue that new data-driven market and business models in the animal health industry must be developed in collaboration with all stakeholders along the food-animal production chain. These new business models should empower small-scale food-animal production businesses to commit and be rewarded for streamlined and near real-time digital data capture. This approach will deliver critical information to fill gaps that currently exist in our understanding of emerging diseases and antimicrobial resistance in production animals and will promote the accessibility of this valuable information to science and society, eventually leading to effective evidence-based policies.

## Author Contributions

Both FV and AT conceptualized and wrote this Perspective paper.

## Conflict of Interest Statement

FV received funding from the OECD’s Co-operative Research Programme on Biological Resource Management for Sustainable Agricultural Systems to attend and present work at the AHEAD 2017 workshop. The second author declares that the research was conducted in the absence of any commercial or financial relationships that could be construed as a potential conflict of interest.

## Footnotes

^1^http://www.datalinker.org.nz/.

^2^http://195.37.233.20/about/.

^3^https://www.tableau.com.

^4^https://www.ibm.com/watson/.

^5^http://research-information.bristol.ac.uk/en/projects/datashield(bfbb6ff5-8717-4085-a922-90795380d442).html.

^6^http://eur-lex.europa.eu/legal-content/EN/TXT/HTML/?uri=OJ:L:2016:084:FULL&from=EN.
